# Be(a)ware of Leukocytosis in Papillary Thyroid Cancer

**DOI:** 10.1155/2022/5799432

**Published:** 2022-07-16

**Authors:** Styliani Laskou, Konstantinos Sapalidis, Christos Topalidis, Triantafyllia Koletsa, Isaak Kesisoglou

**Affiliations:** ^1^3rd Surgical Department, Aristotle University of Thessaloniki, AHEPA Hospital, Thessaloniki, Greece; ^2^Department of Pathology, Faculty of Medicine, Aristotle University of Thessaloniki, Thessaloniki, Greece

## Abstract

Leukocytosis can be present at any time during various malignancies. A 42-year-old male was admitted to our department for surgical management of his metastatic papillary thyroid cancer. Persistent white blood cell (WBC) elevation with left shift led to a thorough investigation. Having excluded other causes, leukocytosis was attributed to thyroid cancer itself. Positive immunostaining for IL-6 and CEA, as well as elevated serum levels, established this connection.

## 1. Introduction

Tumor-related leukocytosis is associated with advanced tumor-stage and poor patient outcomes. However, it has been largely ignored or misinterpreted. If the underlying malignancy is not clinically evident, leukocytosis could be attributed to myeloproliferative neoplasms, misleading the patient's management. Solid tumors such as lung, genitourinary, gastrointestinal, melanoma, and head and neck may secrete cytokines, mainly granulocyte colony-stimulating factor (G-CSF), granulocyte-macrophage CSF (GM-CSF), and/or interleukins. As far as thyroid cancer is concerned, squamous and anaplastic cancer cases associated with leukocytosis have been reported. We report the rare case of a well-differentiated papillary thyroid carcinoma linked to neoplastic leukocytosis and elevated CEA levels.

## 2. Case Report/Case Presentation

In February 2021, a 42-year-old male was admitted to our surgical department due to metastatic papillary thyroid carcinoma detected on an outpatient investigation. He had previously visited a maxillofacial surgeon due to palpable right neck lymphadenopathy, who suggested a neck ultrasound (US). The US showed a 21 × 12 mm hypoechoic solid lesion in the thyroid isthmus and a 33 × 32 × 25 mm lesion with abnormal parenchymal composition and calcifications occupying the right lobe. Central and right lateral cervical lymph nodes were highly suspicious. Fine needle aspiration of the two tumors and a region III lymph node suggested the presence of a metastatic multifocal papillary thyroid carcinoma (PTC).

Thyroid function examination and anti-thyroglobulin antibodies were within the normal range while serum thyroglobulin was 176.9 ng/ml (normal range: 0–50 ng/ml). It was noteworthy that his laboratory tests showed leukocytosis with neutrophilia (WBC: 21,390 K/*μ*l, ΝΕ: 18,300 K/*μ*l), although further investigation was not considered. The patient underwent thyroidectomy with central and right lateral selective lymph node dissection. Due to the aggressive behavior of the tumor, the right thyroid lobe and specimens from the trachea were sent for intraoperative histologic examination, which confirmed the presence of PTC in the right lobe and neoplastic infiltration of the fibroadipose tissue from the trachea along with a metastatic lymph node. The operation was completed, and the patient had an uneventful recovery.

**Figure 1:**
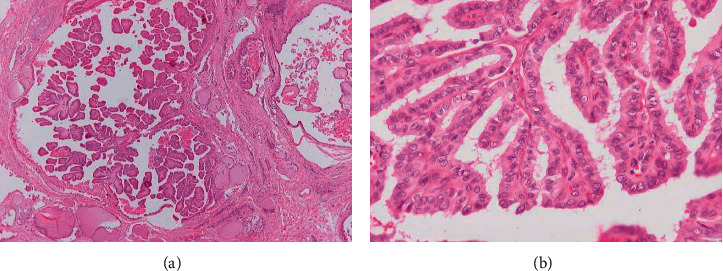
(a) (×40) Papillary structures typical of papillary thyroid carcinoma filling cystic spaces. (b) (×400) Papillary structures are lined by medium-sized cells with eosinophilic cytoplasm and nuclear features typical of papillary thyroid carcinoma.

Gross examination of the thyroid specimen revealed multiple white to tan nodules scattered throughout the gland, both in the lobes, but also in the isthmus, measured from 0.2 cm to 5 cm in diameter. Hematoxylin and eosin-stained sections showed that the described lesions correspond to PTC characterized mainly by papillary structures “(shown in Figure 1(a))”. The lining epithelium consisted of medium-sized cells with eosinophilic cytoplasm and distinctive nuclear features, namely, nuclear overlapping, chromatin clearing, irregular nuclear contour, and nuclear grooves and pseudoinclusions “(shown in Figure 1(b))”. Psammoma bodies were also present in the adjacent area. Stroma was fibrous with dense collagenous with sparsely inflammatory cells. Despite the multiple sections, areas of low differentiation or anaplastic features were not observed. Cancer cells invaded the thyroid capsule and expanded in the surrounding tissues. In addition, seven cervical lymph nodes displayed metastases from the carcinoma. Histologic findings were consistent with a multifocal PTC of the classic variant pT4aN1b according to the pTNM classification scheme.

The patient was reevaluated on the second postoperative month. The US showed left lateral cervical lymphadenopathy and a recurrence of 24 × 19 × 22 mm in the central region despite the meticulous operation. Cervical computed tomography (CT) depicted the aforementioned pathologic lymph nodes. Chest and abdominal CT did not reveal further metastatic lesions. TSH and TG were elevated (18,1 *μ*IU/ml and 131 ng/ml, respectively) while leukocytosis with neutrophilia (WBC: 23,720 K/*μ*l, ΝΕ: 19,800 K/*μ*l) remained. Blasts and immature white blood cells were not found, while erythrocyte sedimentation rate (ESR), C-reactive protein (CRP), and carcinoembryonic antigen (CEA) levels were elevated (22 mm/L, 16 ng/ml, and 18 ng/ml, respectively). After a thorough investigation by the internal medicine department, infectious diseases, leukemia, and myeloproliferative neoplasms were excluded. Searching for the cause of leukocytosis, serum concentrations of granulocyte colony-stimulating factor (G-CSF) and IL-6 were measured. ΙL-6 levels were found elevated (71 pg/ml, normal range<7), which partially explained the observed leukocytosis.

**Figure 2:**
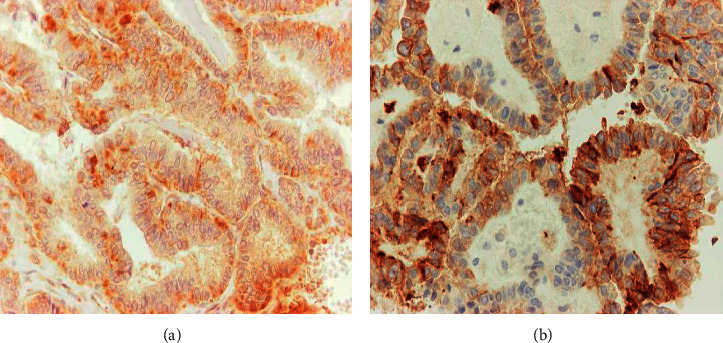
(a) (×400) Immunohistochemical stain for IL-6 proved to be positive in cancer cells. (b) (×400) A small proportion of cancer cells displayed immunoreactivity for CEA.

In June, the patient underwent a second operation with left lateral neck dissection and excision of the recurrence in the central compartment. Histologic examination revealed nine metastatic lymph nodes. After being informed of high serum IL-6 levels, immunohistochemistry with IL-6 antibody was performed, which revealed positivity in a few neoplastic cells “(shown in Figure 2(a))”. Few cells were immunoreactive to CEA antibody as well “(shown in Figure 2(b))”.

Postoperatively, he received a dose of 120 mCi iodine 131. Whole-body scan showed foci of uptake in the thyroid bed. Appropriate thyroxine suppressive therapy was added and on the second post-iodine month patient new examinations were ordered revealing normalized levels of TSH and TG (0,8 *μ*IU/ML and 0.1 ng/ml respectively). Surprisingly, WBC and neutrophils, ESR, CRP, and CEA were also within the normal range (WBC: 9800 K/*μ*l, ΝΕ: 5,000 K/*μ*l, ESR:12, CRP:0,5, CEA: 1,2). IL-6 levels were also measured (2,8 pg/ml). The patient remains under close endocrinologic and surgical surveillance without evidence of recurrence.

## 3. Discussion

This patient may represent the first established classic papillary thyroid carcinoma case with associated leukocytosis and elevated CEA levels caused by IL-6 and CEA expression by tumor cells. Leukocytosis and malignancy have long been linked and known as paraneoplastic leukemoid reaction [1]. In its formal definition, leukemoid reaction refers to persistent leukocytosis (WBC >40 000/*μ*L) in the absence of a hematologic malignancy. Various infections, intoxications, malignancies, severe hemorrhage, or acute hemolysis may be the etiology [2]. Paraneoplastic leukemoid reaction occurs in the presence of a nonhematolymphoid cytokine-secreting tumor and in the absence of bone marrow infiltration by that tumor [3]. In many malignancies, a less profound leukocytosis may be observed, although.

It seems that irregular cytokine production by tumor cells, such as granulocyte colony-stimulating factor (G-CSF), granulocyte-macrophage colony-stimulating factor (GM-CSF), and interleukins (IL-3, IL-6, and TNF-*α*) initiate the pathogenesis [4] leading to granulocytosis. This ability may be present along with tumor development or secondarily after dedifferentiation and can also be seen in metastatic sites even if it is absent in the primary tumor [5]. Cytokine-producing tumor foci grow faster than the nonproducing ones due to an autocrine growth induction phenomenon [6]. In addition to stimulating bone marrow granulocytosis, an inhibition of myeloid cell differentiation in the tumor microenvironment may be observed, causing the accumulation of immature myeloid cells. These myeloid-derived suppressor cells “protect” the tumor, contributing also to neoangiogenesis and cancer progression. Such patients seem to have a poor prognosis [3].

A variety of tumors may be associated with elevated WBC. Tumor-related leukocytosis may be present at any time during the disease, simultaneously, late, or even prior to the diagnosis. Although lung and kidney malignancies are commonly associated with leukocytosis, gastrointestinal, hepatobiliary, genitourinary, melanoma, and head and neck cancers [2] have also been reported.

Thyroid malignancies have also been associated with paraneoplastic leukocytosis. Two cases of squamous cell carcinoma [7, 8] and seventeen cases of anaplastic tumor have been reported [9–23]. Both cases of squamous cell carcinoma were related to marked leukocytosis and hypercalcemia. The authors proposed that tumor cells produced G-CSF, IL- 1a, and PTH-rP, suggesting the existence of a new paraneoplastic syndrome. Elevated G-CSF levels were detected in anaplastic thyroid cancer cases. Only a few case reports evaluated IL-6 levels as either elevated or within the normal range [10–12, 23].

A unique case of aggressive papillary thyroid cancer associated with neutrophilia has been published. Vassilatou et al. hypothesized that the presence of CSF-producing tumor was indicated by elevated GM-CSF serum levels. Bone marrow biopsy revealed infiltration by the papillary thyroid carcinoma [24]. Our patient differed from the above since the tumor cells synthesized and secreted IL-6, which seemed to be the cause of leukocytosis. Perhaps, both cases indicate papillary thyroid cancer as a precursor of anaplastic.

Moreover, tumor cells were positive for CEA with elevated CEA serum levels. CEA is produced as an expression of CEA-related cell adhesion molecule 5 gene and is found in 90% of gastrointestinal cancers, 70% of lung cancers, and 50% of breast cancers [25]. Elevated levels may also be seen in ovarian cancer, appendix, mucinous cystadenoma, and medullary thyroid carcinoma. Since only single cases of CEA-positive papillary thyroid cancer have been observed, its significance remains uncertain.

## 4. Conclusion

Tumor-related leukocytosis is associated with tumor aggressiveness and probably poor prognosis. When dealing with patients with unexplained leukocytosis, a high level of suspicion is required for this entity.

## Data Availability

The datasets used during the current study are available from the corresponding author on reasonable request.

## Additional Points

Tumor-related leukocytosis could represent a form of paraneoplastic syndrome caused by cytokine secretion from tumor cells.
